# Socio-economic factors affecting high infant and child mortality rates in selected African countries: does globalisation play any role?

**DOI:** 10.1186/s12992-022-00855-z

**Published:** 2022-07-07

**Authors:** Mohammad Mafizur Rahman, Khosrul Alam, Rasheda Khanam

**Affiliations:** 1 School of Business, Centre for Health Research, University of Southern Queensland, Toowoomba, Australia; 2 Department of Economics, Bangabandhu Sheikh Mujibur Rahman Science and Technology University, Gopalganj, 8100 Bangladesh

**Keywords:** Infant mortality rate, Child mortality rate, Globalization, Panel data, African countries

## Abstract

**Background:**

Despite the declining trends worldwide, infant and child mortality rates are still high in many African countries. These high rates are problematic; therefore, this study attempts to explore the contributing factors that cause high infant and child mortality rates in 14 African countries using panel data for the period of 2000–2018. In particular, the role globalisation is explored.

**Methods:**

The panel corrected standard error (PCSE), the Feasible generalized least square (FGLS) models, and the pair-wise Granger causality test have been applied as methodological approaches.

**Results:**

The public health expenditure, numbers of physicians, globalization, economic development, education, good governance, and HIV prevalence rate have been revealed as the determinants of infant and child mortality in these countries. All these variables except the HIV prevalence rate negatively affect the infant and child mortality rates, while the HIV prevalence rate is found to be positive. Bidirectional and unidirectional causal relationships between the variables are also attained.

**Conclusions:**

Effective socio-economic policy priority with due consideration of globalization should be emphasized to reduce infant and child mortality rates in these countries.

## Background

Globally, child and infant mortality has been declining since the mid-1980s with a greater decline throughout the 1990s [89] due to the combined effects of economic development, progress in medical science and technologies, improvements in the socio-economic environment, and health interventions [90]. However, this progress is not even, and for some African countries infant and child mortality rates are still alarmingly high. According to the WDI [87], child mortality in Chad is 113.8 per 1000 live births and in Nigeria and Somalia, it is 117. Infant mortality in Somalia is 89.5 per 1000 live births [18]. With the dark shadows of the poverty, these African countries suffer from a number of socio-economic difficulties such as lack of adequate government health expenditure, insufficient numbers of physicians, existence of higher rate of HIV/AIDS, lack of utilization of globalization facilities, inadequate education and poor economic development which may be the major contributory factors for the high infant and child mortality rates. To the governments of these countries, reduction of infant and child mortality rates is a great challenge to achieve the Sustainable Development Goals (SDGs) declared in 2015. The major goal is to reduce infant mortality rate to at least as low as 12 per 1000 live births, and child mortality rate to at least as low as 25 per 1000 live births by 2030 (Goal-3 of SDGs [84, 85]). The on-going COVID-19 pandemic has also made infant and child health more vulnerable, and this needs urgent and efficient attention from policy makers to articulate effective strategies.

The present study is an endeavour to probe the socio-economic and globalization factors leading to high infant and child mortality rates in the selected 14 African countries (full list is in section- 3.1). The countries have been selected based on the ranking of the highest mortality rates and availability of data. These countries have a total population of 425.131 million and the total GDP is US$726.249 billion, which is just 0.840% of the world’s GDP [87]. The average infant mortality rate in this region in 2018 was 38.121 per 1000 live births, and the highest was in Mali (61.800 per 1000 live births). Similarly, the average child mortality rate in this area in 2018 was 53.993 per 1000 live births, and the highest was also experienced in Mali (97.400 per 1000 live births). The infant and child mortality rates in the studied areas are much higher compared to other developed regions, for example the OECD, EU, and North America, where infant mortalities are 6.083, 3.380, and 5.500 (per 1000 live births) and child mortalities are 7.158, 4.032, and 6.400 (per 1000 live births), respectively [87]. The average public health expenditures, physicians, HIV prevalence rates, globalization index, and expected years of schooling are 1.975%, 0.519 (per 1000 people), 4.250%, 54.917, and 11.450 years, respectively [32, 87] reflecting poor indicators. Therefore, more intensive consideration of infant and child health is essential.

Some studies (see [2, 3, 10, 28, 33, 43, 62–64, 78]) have attempted to investigate the determinants of infant and child mortality. However, unanimous, inclusive, and convincing policy outcomes are not properly perceived, especially for the mentioned African countries (see section- 3.1). Therefore, this study aims to fill up the gaps in the literature, by investigating the influences of public health expenditures, numbers of physicians, HIV prevalence rate, globalization, economic growth, and education on infant and child mortality rates in the selected African countries. In addition, this study will identify the causality of infant and child mortality rates with these socio-economic factors.

This study contributes to the existing literature in the following ways. First, this is the first study in the literature, to the best of our knowledge, which investigates the role of public health expenditures, physicians, HIV prevalence rate, globalization, economic growth, and education on infant and child mortality rates in the context of selected African countries. Second, this study uses the most updated available data of 19 years (2000–2018) for the selected variables. Third, the findings are achieved by adopting sophisticated econometric methods: cross-sectional dependence test, Modified Wald test, Wooldridge test, the Panel corrected standard error (PCSE) model, the Feasible generalized least square (FGLS) model, and the pair-wise Granger causality test. Fourth, the outcomes will provide unique and novel guidelines for policy makers to reduce infant and child mortality rates by enunciating public health expenditures, physicians, HIV prevalence rate, globalization, economic growth, and education policies.

The study is arranged in the following order: following the introduction, section 2 displays literature review; section 3 mentions methods; section 4 describes the results; section 5 presents the discussions; and section 6 provides the conclusion and policy implications.

## Literature review

Among the extant literature, some studies (see, for example [3, 10, 19, 43, 61–64, 66]) investigated the effects of public health expenditure on infant and child mortality rates in the context of African regions These studies found that public health expenditure significantly reduced infant and child mortality rates in African regions. Conversely, Akinlo and Sulola [3] revealed that government health expenditure increased infant and child mortality in sub-Saharan Africa due to the high level of corruption of public health expenditure. In a single country perspective, Edeme [21] showed that the public health expenditure had reducing effect on infant mortality rate in Nigeria for the data period of 1981–2014. Similar findings were also observed by Manda et al. [53] for Kenya, Boachie and Ramu [16] for Ghana, Akinkugbe and Mohanoe [2] for Lesotho, and Makochekanwa and Madziwa [52] for Zimbabwe. But, Hlafa et al. [33] obtained varying effects of public health expenditure on child mortality rate depending on provincial management and infrastructure availability across 9 provinces of South Africa during the data period of 2002–2016. The role of public health expenditure on infant and child mortality rates has also been explored beyond African regions, notably in the works of Farag et al. [24] for133 low and middle-income countries, Rahman et al. [72] for SAARC-ASEAN regions, Akinci et al. [1] for MENA region, and Golinelli et al. [29] for Italy. These investigations are useful but the role of public health expenditure on infant and child mortality rates requires further investigation.

The role of physicians on reducing infant and child mortality is also investigated in the literature on African countries (see [2, 10, 33]). Anyanwu and Erhijakpor [10] revealed a negative association between the number of physicians, and infant and child mortality rates in 47 African countries over the period of 1999–2004. Similar results were also observed by Akinkugbe and Mohanoe [2] and Hlafa et al. [33] in Lesotho and South Africa, respectively. Beyond Africa, the importance of the number of physicians on infant and child mortality rates is also noted in the studies of Jebeli et al. [37] for 26 OECD countries, Muldoon et al. [57] for 136 UN member countries, Farahani et al. [25] for global contexts, Liebert and Mäder [50] for Germany, Russo et al. [77] for Brazil, and Shetty and Shetty [81] for Asian countries. However, Oloo [67] revealed that the proportion of doctors for every 100,000 population had an insignificant impact on child mortality rates in developing countries. Therefore, the role of physicians should be critically assessed for ensuring good child health.

HIV/AIDS has had a devastating effect on infant/child mortality in African countries, which is also observed in the contemporary works of Anyanwu and Erhijakpor [10] Garrib et al. [28] Kiross et al. [43]; and Salahuddin et al. [78]. In this connection, Anyanwu and Erhijakpor [10] revealed the positive impact of HIV prevalence on infant and child mortality rates in the 47 African countries. Garrib et al. [28] revealed that HIV/AIDS was the single largest cause of higher infant and child mortality rates in a rural area of South Africa from the community-based survey data of 2000–2002. Similar results were also obtained by Preble [70] for 10 Central and East African countries, Kiross et al. [43] for 46 sub-Saharan African countries, and Salahuddin et al. [78] for South Africa. In the same way, Arunda et al. [7] observed that a mother’s HIV status was positively associated with the child mortality rate in Tanzania during 2003–2012. The same results were also experienced by Lallemant et al. [46] for the Republic of Congo, Tlou et al. [82] for rural South Africa, Nakiyingi et al. (2003) [58] for Uganda, and Marinda et al. [54] for Zimbabwe. However, Akinlo and Sulola [3] established a significant negative relationship between HIV prevalence and infant and child mortality rates in 10 sub-Saharan Africa. Novignon et al. [62] found an indeterminate impact of HIV prevalence rate on infant mortality in sub-Saharan African countries. Other than Africa, Oloo [67] discerned that that the rate of HIV prevalence increases child mortality in developing countries. Therefore, the impact of HIV prevalence also requires careful examination.

Economic growth also significantly affects infant and child health, where many of the researchers noted that economic growth reduced infant and child mortality rates in African nations (see [3, 8, 62, 78]; and [43]). Beyond African countries, many of the researchers observed that economic growth had a statistically significant impact on reducing the infant and child mortality rates (see [20, 34, 72]; and [74]). However, Pérez-Moreno et al. [68] identified that a decrease in GDP per capita causes a significant increase in child mortality, whereas the rise does not affect child mortality significantly in the Least Developed Countries (LDCs). Therefore, further thorough inspection of the role of economic growth on infant and child health is needed.

Education has a significant role in the reduction of infant and child mortality rates by creating public awareness and knowledge about healthy life. The important role of education in reducing infant and child mortality has been found in some contemporary literature based on African countries (see [2, 9, 39, 42, 43, 93]). Beyond African countries, many writers found that there was a negative relationship between the child mortality rate and the education rate (see [26, 27, 45, 56]; and [74]). However, some writers perceived no statistically significant effect of education on infant and neonatal mortality rates (see [25], and [5]). Therefore, more enquiries regarding the role of education on infant and child mortality rate are justified.

Globalization is now considered as another important element in affecting child and infant health in the African countries (see [60, 80, 94]). Nguea et al. [60] obtained that the globalization reduced infant and child mortality rate in 32 sub-Saharan African countries. Shahbaz et al. [80] identified that the globalization has positive impact on life expectancy at birth in the 16 sub-Saharan African countries. Conversely, Young et al. [94] observed that the globalization increases the risk of diabetes and cardiovascular disease that contribute to worse health outcomes in sub-Saharan African countries. Other than African countries, a few studies also found the significant influences of globalization on infant and child health in other parts of the world. Olagunju et al. [65] found that the globalization significantly reduced child mortality rate during the data period of 1970–2015 in 110 developing countries. Similarly, Welander et al. [91] and Jani et al. [36] observed that the globalization diminished infant mortality rate in 70 developing countries, and global context, respectively. Martens et al. [55] identified that the globalization reduced both infant and child mortality rate in case of 117 countries. Thus, the role of globalization needs to be critically inspected for ensuring better health outcomes for infants and children.

After inspecting the above literature meticulously, it has been found that the earlier evidence on the role of causative factors on infant and child mortality rates is inconclusive. As a result, it is difficult for policy makers to articulate well-suited policies to resolve the problem of high infant and child mortality rates. Moreover, a study that simultaneously examines the effects of socio-economic factors (e.g. GDP, education, public health expenditures,) health facilities factors (number of physicians, and HIV prevalence rate), and globalization on infant and child mortality rates has been very limited in past literature, especially in the African region. Therefore, this study attempts to fill-up this gap in the literature and provides policy recommendations to reduce infant and child mortality rates.

## Methods

### Variables and data

In this study we have used the infant mortality (INF) rate and child mortality (CM) rate as our dependent variables. Infant mortality rate is defined as the number of infants dying before reaching 1 year per 1000 live births, whereas the child mortality rate is considered as the probability of newborn babies dying before reaching 5 years per 1000 live births in a provided year. The independent variables are: public health expenditure (PUH) as a percentage of GDP; physicians^1^ (PHY) as the number of physicians per 1000 people; HIV prevalence rate as percentage of the population ages 15–49 years living with HIV; gross domestic product (GDP) as current US$ as a proxy of economic development; education^2^ (EDU) considered as the expected years of schooling; and the KOF Globalisation Index, which is used to measures the economic, social and political dimensions of globalisation of a country over a longer period of time; it is a composite index on a scale of 1–100, where 1 denotes the least and 100 denotes the most globalised. Due to the lack of data for all countries in the studied region, we had to use expected year of schooling as a proxy of education rather than using literacy rate, school enrolment or completion rate. Furthermore, we have included good governance (GG) as a control variable, which is calculated from the average rank of six vital indicators as government effectiveness, voice and accountability, political stability and absence of violence/terrorism, rule of law, control of corruption, and regulatory quality. The rationale for selecting these variables is as follows. First, public health expenditures usually facilitate essential medical facilities and treatments at a cheaper rate which are affordable by the underprivileged people, which in turn, improve the health of infants and children. Second, physicians may guide and prescribe the use of effective and proper medication that will improve health conditions and may reduce infant and child mortality rates. Third, HIV prevalence rate increases the chances of infants and children being affected and increase death rates. Fourth, economic growth guarantees healthier living standards and more medical amenities for combating infant and child mortalities. Fifth, education imparts knowledge and awareness to handle infant and child health in a better way. Sixth, globalization ensures better and modern amenities that reduce infant and child mortality rate.

We have used the balanced panel data of 2000–2018. Annual data are collected from the World Development Indicators [87], World Bank for the variables of infant mortality rate, child mortality rate, public health expenditure, physicians, and GDP. The good governance index data are collected from World Governance Indicator [88] of World Bank. The data of HIV and education are collected from Human Development Reports [32] of the United Nation Development Program (UNDP). The globalization index is obtained from KOF Globalisation Index data [44]. Table 1 below shows the details of data and variables used. Some missing data of HIV prevalence rate and physicians’ rate are linearly interpolated through E-views-11. To perform the estimation, two renowned statistical software packages, STATA-16 and E-views-11 are used. Due to the lack of data on the selected variables we had to limit this research to 14 African countries. The selected countries are Algeria, Benin, Egypt, Ethiopia, Kenya, Malawi, Mali, Mauritania, Mauritius, Mozambique, Namibia, Rwanda, Togo, and Zambia.

**Table 1:** Details of data and variables

Variables	Symbols	Measurements	Data sources
Infant mortality rate	INF	Number of infants dying before reaching one year per 1000 live births	World Development Indicators [87]
Child mortality rate	CM	Probability of newborn babies dying before reaching five years per 1000 live births in a provided year	World Development Indicators [87]
Public health expenditure	PUH	Percentage of GDP	World Development Indicators [87]
Number of physician	PHY	Number of physicians per 1000 people	World Development Indicators [87]
HIV prevalence rate	HIV	Percentage of the population ages 15–49 years living with HIV	Human Development Reports [32]
Gross domestic product	GDP	Current US$	World Development Indicators [87]
Education	EDU	The expected years of schooling	Human Development Reports [32]
Good governance	GG	Average rank of six vital indicators as government effectiveness, voice and accountability, political stability and absence of violence/terrorism, rule of law, control of corruption, and regulatory quality	World Governance Indicators [88]
Globalization	GLOB	Measures of the economic, social and political dimensions of globalisation of a country over a longer period of time which is a composite index on a scale of 1–100, where 1 denotes the least and 100 denotes the most globalised	KOF Globalisation Index [44]

### Theory and econometric approach

Becker’s [15] model of human capital considers medical care as an important element of human capital development. Subsequently Grossman’s [31] model of health demand views health as a durable capital stock that produces healthy time. However, over time, the initial stock of health of a person depreciates [31]; therefore, there is a need of investment to maintain this health capital to a certain level. Higher infant and child mortality rates indicate vulnerable public health condition of the countries, and deter the build-up of efficient human capital, which ask proper attention to control these through the effective care of driving factors [12] like proper investment in the health sector.. In the household setting, individuals are responsible for this investment, while in a macro setting, government is responsible for proper investment to maintain its nation’s health capital. Government investments are linked with public health expenditure, physicians, HIV prevalence rate, globalization, economic development, and education among others. This study investigates the role of these factors in determining the infant and child mortality rate under the notion of human capital model.

While the justification for selecting the independent variables is noted in section 3.1, it can be further extended that these variables are chosen following the past literature. For example, we have used infant mortality rate following Araujo et al. [6] and Urquieta-Salomón et al. [83], child mortality rate following Rahman and Alam [74] and Wang [86], public health expenditure following Wang [86], Rahman et al. [72], and Rahman and Alam [75], physicians rate following Russo et al. [77], Rahman et al. [73], and Karyani et al. [40], HIV prevalence rate following Newell et al. [59], Novignon et al. [62] and Kiross et al. [43], globalization following Olagunju et al. [65] and Welander et al. [91], GDP following Ensor et al. [23] and Liang et al. [49], education following Kiross et al. [43] and Rahman and Alam [74], and good governance following Farag et al. [24] and Emamgholipour and Asemane [22].

Therefore, the used models in this study are:

1 $$\mathrm{INF}=\mathrm{f}\ \left(\mathrm{PUH},\mathrm{PHY},\mathrm{HIV},\mathrm{GDP},\mathrm{EDU},\mathrm{GG},\mathrm{GLOB}\right)$$

2 $$\mathrm{CM}=\mathrm{f}\ \left(\mathrm{PUH},\mathrm{PHY},\mathrm{HIV},\mathrm{GDP},\mathrm{EDU},\mathrm{GG},\mathrm{GLOB}\right)$$

Where, INF, CM, PUH, PHY, HIV, GDP, EDU, GG, GLOB represent infant mortality rate, child mortality rate, public health expenditure, number of physician, HIV prevalence rate, gross domestic product, education, good governance, globalization, respectively. Thus, after converting all the variables of Eqs. (1) and (2) into natural logarithmic form, we obtain the following equations where coefficients of each variable directly provide the elasticity [73, 75]:

3 $${\mathrm{LNINF}}_{\mathrm{t}}=\upalpha +{\upbeta}_1{\mathrm{LNPUH}}_{\mathrm{t}}+{\upbeta}_2{\mathrm{LNPHY}}_{\mathrm{t}}+{\upbeta}_3{\mathrm{LNGLOB}}_{\mathrm{t}}+{\upbeta}_4{\mathrm{LNIMM}}_{\mathrm{t}}+{\upbeta}_5{\mathrm{LNGDP}}_{\mathrm{t}}+{\upbeta}_6{\mathrm{LNEDU}}_{\mathrm{t}}+{\upbeta}_7{\mathrm{LNGG}}_{\mathrm{t}}+{\varepsilon}_t$$

4 $${\mathrm{LNCM}}_{\mathrm{t}}=\upalpha +{\upbeta}_1{\mathrm{LNPUH}}_{\mathrm{t}}+{\upbeta}_2{\mathrm{LNPHY}}_{\mathrm{t}}+{\upbeta}_3{\mathrm{LNHIV}}_{\mathrm{t}}+{\upbeta}_4{\mathrm{LNGLOB}}_{\mathrm{t}}+{\upbeta}_5{\mathrm{LNGDP}}_{\mathrm{t}}+{\upbeta}_6{\mathrm{LNEDU}}_{\mathrm{t}}+{\upbeta}_7{\mathrm{LNGG}}_{\mathrm{t}}+{\varepsilon}_t$$

Hence, α is the intercept, and β_1_, β_2_, β_3_, β_4_, β_5_, β_6,_ β_7_ are coefficients and ε_t_ is the error term.

Due to the influence of economic globalization, there may exist similarities and dependencies in the panel countries irrespective of size, culture, and population, which can increase the probability of heteroscedasticity, serial correlations and cross-sectional dependences (CD) among the variables. To detect the heteroscedasticity, the Modified Wald statistic for groupwise heteroskedasticity will be used following the methodology of Baum [13] and to check the existence of serial correlation, the Wooldridge test for autocorrelation in panel data will be employed as per the methodology of Wooldridge [92]. In this study, the Pesaran [69] CD statistic test will be used to diagnose cross-sectional dependence among the variables under following equation:

5 $$CD=\sqrt{\frac{2T}{N\left(N-1\right)}}\ \left({\sum}_{i=1}^{N-1}{\sum}_{j=i+1}^N\sqrt{T_{ij}{\hat{p_{ij}}}^2}\right)$$

Here, T shows time, N shows cross-sectional dimension and ${\hat{p_{ij}}}^2$ denotes the pairwise cross-sectional correlation coefficient of residuals. The decision rule is that the null hypothesis has cross-sectional independence with CD ^~^ *N* (0, 1) and the alternative hypothesis has cross-sectional dependence.

As the existence of heteroscedasticity, autocorrelation and cross-sectional dependency in the panel data may offer inefficient statistical inference on the model analysis, standard fixed effect model may not produce robust outcomes [51, 71]. Hence this study utilizes the panel corrected standard error (PCSE) model following the methodology of Beck and Katz [14]. In the case of small panels and finite sample bias along with heteroscdasticity, serial correlation, and cross sectional dependency, the panel corrected standard error (PCSE) model generates appropriate outcomes [17]. To check the robustness of the estimated results the feasible generalized least square (FGLS) model is used in line with the methodology of Le and Nguyen [47], Ikpesu et al. [35], and Alonso et al. [4], as this is also appropriate in addressing the heteroscdasticity, serial correlation, and cross sectional dependency issues.

In the current study a pair-wise Granger [30] causality of stacked test will be adopted to identify the causality among the variables following the methodologies of Revathy and Paramasivam [76] and Seitaridis and Koulakiotis [79] as under:

6 $${\mathrm{Y}}_{\mathrm{i},\mathrm{t}}={\uppi}_{0,\mathrm{i}}+{\uppi}_{1,\mathrm{i}}{\mathrm{Y}}_{\mathrm{i},\mathrm{t}-1}+\dots \dots +{\uppi}_{\mathrm{k},\mathrm{i}}{\mathrm{Y}}_{\mathrm{i},\mathrm{t}-1}+{\updelta}_{1,\mathrm{i}}{\mathrm{X}}_{\mathrm{i},\mathrm{t}-1}+{\upvartheta}_{\mathrm{i},\mathrm{t}}$$

7 $${\mathrm{X}}_{\mathrm{i},\mathrm{t}}={\uppi}_{0,\mathrm{i}}+{\uppi}_{1,\mathrm{i}}{\mathrm{X}}_{\mathrm{i},\mathrm{t}-1}+\dots \dots +{\uppi}_{\mathrm{k},\mathrm{i}}{\mathrm{X}}_{\mathrm{i},\mathrm{t}-1}+{\updelta}_{1,\mathrm{i}}{\mathrm{Y}}_{\mathrm{i},\mathrm{t}-1}+{\upvartheta}_{\mathrm{i},\mathrm{t}}$$

Where, t and i indicate the time period dimension and the cross-sectional dimension of the panel. The stacked causality test reflects common values of all coefficients across the all cross-sections level,which can be written as under:

8 $${\uppi}_{0,\mathrm{i}}={\uppi}_{0,\mathrm{j}},{\uppi}_{1,\mathrm{i}}={\uppi}_{1,\mathrm{j}},\dots \dots \dots .,{\uppi}_{\mathrm{k},\mathrm{i}}={\uppi}_{\mathrm{k},\mathrm{j}},\forall \mathrm{i},\mathrm{j}$$

9 $${\updelta}_{0,\mathrm{i}}={\updelta}_{0,\mathrm{j}},{\updelta}_{1,\mathrm{i}}={\updelta}_{1,\mathrm{j}},\dots \dots \dots .,{\updelta}_{\mathrm{k},\mathrm{i}}={\updelta}_{\mathrm{k},\mathrm{j}},\forall \mathrm{i},\mathrm{j}$$

Hence the null hypothesis (H_0_) is considered that, Y does not Granger causes X, and alternative hypothesis (H_1_) is taken as Y Granger causes X. In this case there may arise three outcomes: unidirectional causality, bidirectional causality and no causality.

## Results

The descriptive statistics of the studied variables are presented in Table 2 where the mean values of the natural log of infant and child mortality rates are 3.829 and 4.206 respectively. Similarly, the mean values of the natural log of public health expenditure, physicians, HIV, GDP, education, good governance, and globalization are 0.426, − 1.959, 0.681, 23.283, 2.304, 3.527, and 3.911, respectively. In the same way the median, maximum and minimum values, standard deviation, skewness, kurtosis, Jarque-Bera and probability values are depicted in Table 2. All the outcomes efficiently determine the robustness of the estimation.

**Table 2:** Descriptive statistics

Description	LNINF	LNCM	LNPUH	LNPHY	LNHIV	LNGDP	LNEDU	LNGG	LNGLOB
Mean	3.830	4.206	0.426	−1.959	0.681	23.283	2.304	3.527	3.911
Median	3.960	4.369	0.442	−2.148	0.854	23.026	2.380	3.554	3.917
Maximum	4.718	5.232	1.663	1.040	2.785	26.530	2.728	4.350	4.282
Minimum	2.526	2.674	−0.964	−4.510	−2.303	21.116	1.459	2.615	3.379
Std. Dev.	0.533	0.651	0.563	1.471	1.635	1.293	0.251	0.403	0.176
Skewness	−0.805	−0.810	−0.009	0.449	−0.535	0.790	− 0.845	0.251	− 0.224
Kurtosis	2.964	2.808	2.799	2.124	2.195	2.880	3.403	2.282	3.356
Jarque-Bera	28.732	29.491	0.450	17.463	19.873	27.813	33.453	8.502	3.628
Probability	0.000	0.000	0.798	0.000	0.000	0.000	0.000	0.014	0.163
Sum	1018.746	1118.825	113.364	− 521.196	181.235	6193.271	612.847	938.179	1040.340
Sum Sq. Dev.	75.19134	112.1454	84.027	573.461	708.470	442.852	16.707	43.079	8.213
Observations	266	266	266	266	266	266	266	266	266

All the variables are converted into the natural logarithm form

Table 3 shows the findings of heteroscedasticity and autocorrelation tests. Test statistics and their corresponding probabilities show that there is the existence of heteroscedasticity and autocorrelation in both infant mortality and child mortality models.

**Table 3:** The outcomes of heteroscedasticity and autocorrelation Test

Model	LNINF		LNCM		Presence
Test	Test statistic	***P***-value	Test statistic	***P***-value	
Modified Wald test for group wise heteroscedasticity	χ^2^ = 825.31	0.000	χ^2^ = 1459.35	0.000	Yes
Wooldridge test for autocorrelation in panel data	F-statistic = 174.602	0.000	F-statistic = 198.140	0.000	Yes

Auto correlation: Wooldridge test for autocorrelation in panel data. H0: no first-order autocorrelation

Heteroscedasticity: Modified Wald test for groupwise heteroskedasticity; Ho: sigma(i)^^^ 2 = sigma^^^ 2 for all i: No heteroskedasticity

The results of the cross-sectional dependence test under the Pesaran’s [69] statistic are reported in Table 4. All the outcomes of the Table 3 except good governance (GG) reject the null hypothesis of no cross-sectional independence at 1% level and ensure that there is a significant indication of the existence of cross-sectional dependence. In case of good governance, the value is taken of their rank for estimation, and thus it shows cross-sectional independence.

**Table 4:** The results of Pesaran [69] CD test for cross-sectional dependence

Variables	Statistic	p-value
LNINF	38.964	0.000
LNCM	39.673	0.000
LNPUH	4.11	0.000
LNPHY	15.736	0.000
LNHIV	11.502	0.000
LNGDP	39.277	0.000
LNEDU	35.185	0.000
LNGG	−0.386	0.700
LNGLOB	37.172	0.000

Under the null hypothesis of cross-section independence, CD ~ N(0,1) *P*-values close to zero indicate data are correlated across panel groups

From the above estimations, the existence of heteroscedasticity, autocorrelation, and cross-sectional dependency has been identified. Thus, the PCSE model is suitable that addresses the problems of heteroscedasticity, autocorrelation, and cross-sectional dependency in the context of small panels and short data periods ( [11, 38]; Le et al. [48]). Table 5 depicts the outcomes of PCSE model. In case of both infant mortality and child mortality, the coefficients of public health expenditure are − 0.031 and − 0.033 which are negative and statistically significant at 5% level, respectively. This implies that 1% increase in public health expenditure significantly reduces the infant and child mortality by 0.031 and 0.033%, respectively. Public health expenditures allow poor people to receive more medical facilities at arm’s length. The coefficients of physicians’ rate are (− 0.031 and − 0.034), which are negative and statistically significant at 1% level, meaning that 1% increase in physicians’ rate will reduce the infant and child mortality by 0.031 and 0.034%, respectively. The coefficients of HIV are 0.076 and 0.108 which are positive and significant at 1% level, indicating that 1% increase in HIV prevalence increases the infant mortality rate by 0.076% and child mortality rate by 0.108%. Higher HIV retards immunity of the people and causes the increase of all deaths including infant and child mortalities. Similarly, the coefficients of GDP are − 0.093 and − 0.117 which are also negative and significant at 1% level showing that 1% increase in GDP reduces infant and child mortality rates by 0.093 and 0.117%. Economic growth enhances the capacity to receive medical treatment of infant and child; so this is expected result. In the same way, the coefficients of expected years of schooling (education) are − 0.743 and − 0.850 which are significant at 1% level, displaying that 1% increase in education decreases infant mortality rate by 0.743% and child mortality rate by 0.850%. These results imply that educated couple can take wise and conscious decisions for the betterment of their infant and child health. The coefficients of good governance are − 0.111 and − 0.107 which are also negative and significant at 1% level, denoting that good governance reduces infant and child mortality rates. Good governance makes the strong institutional bodies comprised with transparency and accountability, which ensure better infant and child health facilities. The coefficients of globalization are − 0.502 and − 0.587 which are negative and statistically significant at 1% level, denoting that 1% increase in globalization decreases infant mortality rate by 0.502% and child mortality rate by 0.587%. Globalization offers inter-exchange of medical knowledge and facilities to diagnose diseases and offer treatment globally, which ensure the reduction of the infant and child mortality rates.

**Table 5:** Panel corrected standard error (PCSE) model results

Variables	PCSE regression (LNINF case)	PCSE regression (LNCM case)
LNPUH	−0.023* (− 1.86)	− 0.026* (− 1.76)
LNPHY	−0.018** (− 2.12)	−0.022** (− 2.21)
LNHIV	0.076*** (4.86)	0.109*** (5.89)
LNGDP	−0.084*** (− 4.95)	− 0.102*** (− 5.11)
LNEDU	− 0.530*** (− 7.66)	−0.623*** (− 7.49)
LNGG	− 0.068*** (− 2.60)	−0.070** (− 2.27)
LNGLOB	− 0.502*** (− 5.17)	−0.587*** (− 5.14)
_Constant	9.110*** (21.57)	10.418*** (20.87)
R-squared	0.977	0.974
Wald chi^2^	455.26	492.73
Probability	0.000	0.000
Number of observations	266	266
Number of groups	14	14

***, **, and * denote significance level at 1, 5 and 10%, respectively. Figures in the parentheses are z-statistics

To check the robustness and confirm the results of the PCSE model, we have re-run the models using feasible generalised least square (FGLS) estimation technique. The results (in Table 6) of the FGLS models confirm the results attained by the PCSE models, where all variables are statistically significant at different significance levels except good governance which is insignificant. The FGLS estimates also show that increase in public health expenditure, numbers of physicians, GDP, education, good governance, and globalization reduce infant and child mortality rates, while an increase in the HIV prevalence rate increases both the infant and child mortality rates.

**Table 6:** Feasible generalized least square (FGLS) model results

Variables	FGLS regression (LNINF case)	FGLS regression (LNCM case)
LNPUH	−0.025** (− 2.31)	− 0.028** (− 2.26)
LNPHY	− 0.013* (− 1.74)	−0.015* (− 1.73)
LNHIV	0.063*** (5.41)	0.103*** (7.65)
LNGDP	−0.112*** (− 8.97)	− 0.131*** (− 8.99)
LNEDU	− 0.467*** (− 8.11)	−0.563*** (− 8.25)
LNGG	− 0.029 (− 1.40)	−0.016 (− 0.69)
LNGLOB	− 0.338*** (− 4.43)	−0.386*** (− 4.34)
_Constant	8.905*** (27.12)	10.051*** (26.22)
Wald chi^2^	699.95	842.69
Probability	0.000	0.000
Number of observations	266	266
Number of groups	14	14

***, **, and * denote significance level at 1, 5 and 10%, respectively. Figures in the parentheses are z-statistics

In Table 7, the outcomes of the pair-wise Granger causality test are displayed in F-statistics, along with their probability values. We found a bidirectional causal association between the infant mortality rate and public health expenditure, physician, and globalization, and unidirectional causality running from infant mortality to HIV prevalence rate, and good governance to infant mortality rate. Similarly, a bidirectional causal association of child mortality with public health expenditure, physician, globalization, and HIV prevalence rate, and a unidirectional causality from good governance to child mortality rate is also achieved.

**Table 7:** Causality test results

Null Hypothesis:	F-Stat.	Prob.	Decision
**LNINF Case**			
LNPUH does not cause LNINF	8.939***	0.003	LNPUH ↔ LNINF (bidirectional causality)
LNINF does not cause LNPUH	4.432**	0.036	
LNPHY does not cause LNINF	27.433***	0.000	LNPHY ↔ LNINF (bidirectional causality)
LNINF does not cause LNPHY	5.809**	0.017	
LNHIV does not cause LNINF	2.347	0.1268	LNINF→ LNHIV (unidirectional causality)
LNINF does not cause LNHIV	25.063***	0.000	
LNGDP does not cause LNINF	0.468	0.494	No causality
LNINF does not cause LNGDP	1.795	0.182	
LNEDU does not cause LNINF	1.818	0.179	No causality
LNINF does not cause LNEDU	1.552	0.214	
LNGG does not cause LNINF	5.331**	0.022	LNGG→ LNINF (unidirectional causality)
LNINF does not cause LNGG	1.513	0.220	
LNGLOB does not cause LNINF	32.279***	0.000	LNGLOB ↔ LNINF (bidirectional causality)
LNINF does not cause LNGLOB	14.606***	0.000	
**LNCM Case**			
LNPUH does not cause LNCM	11.329***	0.001	LNPUH ↔ LNCM (bidirectional causality)
LNCM does not cause LNPUH	4.505**	0.035	
LNPHY does not cause LNCM	41.553***	0.000	LNPHY ↔ LNCM (bidirectional causality)
LNCM does not cause LNPHY	6.107**	0.014	
LNHIV does not cause LNCM	8.015***	0.005	LNHIV ↔ LNCM (bidirectional causality)
LNCM does not cause LNHIV	27.553***	0.000	
LNGDP does not cause LNCM	0.592	0.442	No causality
LNCM does not cause LNGDP	2.124	0.146	
LNEDU does not cause LNCM	1.941	0.165	No causality
LNCM does not cause LNEDU	2.612	0.107	
LNGG does not cause LNCM	3.400*	0.066	LNGG→ LNCM (unidirectional causality)
LNCM does not cause LNGG	1.155	0.284	
LNGLOB does not cause LNCM	24.555***	0.000	LNGLOB ↔ LNCM (bidirectional causality)
LNCM does not cause LNGLOB	14.217***	0.000	

^***, **^ and ^*^ denote significance level at 1, 5, and 10%, respectively

## Discussions

The main outcomes provided in Table 5 are validated by the outcomes of Table 6. The reducing role of public health expenditure on infant and child mortality rates is consistent with the findings of Novignon et al. [62], Kiross et al. [43], Chireshe and Ocran [19], Rahman et al. [72], but contradictory with the results of Akinlo and Sulola [3], Hlafa et al. [33]. These results are consistent with the expectation that government expenditure on health is likely to increase and improve medical facilities and makes these accessible to all, which in turn would reduce child and infant mortalities. The negative result of the of physicians per 1000 is in line with the findings of Anyanwu and Erhijakpor [10], Akinkugbe and Mohanoe [2], Hlafa et al. [33], Jebeli et al. [37], Muldoon et al. [57], but not in line with the findings of Oloo [67]. More physicians per 1000 people ensures more medical treatment, consultation facilities and appropriate guidance during critical health conditions, which improves infant and child health. The positive influence of HIV prevalence on infant and child mortality rates is pertinent to the observations of Garrib et al. [28], Preble [70], Kiross et al. [43], Salahuddin et al. [78], but not pertinent to the findings of Akinlo and Sulola [3], and Novignon et al. [62]. The prevalence of HIV intensifies the risk of dying and spoils the immunity system of many people, which act as a catalyst in increasing infant and child mortality rates. The outcome of the globalization variable on infant and child mortality rates is relevant to the results of Nguea et al. [60], and Shahbaz et al. [80], but not consistent with the results of Young et al. [94]. Globalization provides the world-wide facilities and amenities to avail more modern medical equipment, medications, and consciousness to promote infants and children’s health. In case of the negative effect of economic development on infant and child mortality rates the finding is in line with the findings of Salahuddin et al. [78], Akinlo and Sulola [3], Kiross et al. [43], Rahman et al. [72] and Rahman and Alam [74], but not in line with the findings of Pérez-Moreno et al. [68]. The economic development ensures more medical facilities, and instrumental amenities, and provides better living standards which also play a role in reducing infant and child mortalities. In terms of the inverse influence of education, this outcome is consistent with the outcomes of Kiross et al. [43], Kiros and Hogan [42], Keats [41], Alemu [9], Kousar et al. [45]. Education imparts knowledge and builds awareness for maintaining good health, which also plays a role in reducing infant and child mortality rates. The negative impact of good governance on infant and child mortality rates displays the better capacity building, and accountability for ensuring child health. This outcome is in line with Farag et al. [24].

## Conclusions and policy implications

Infant and child mortality rates are higher in the African region compared to the rest of the world. In this paper, we attempt to explore the main determinants of infant and child mortality rates in 14 African countries using data for the period 2000–2018. This is the first macro-study of this nature that investigates the role of public health expenditures, physicians, HIV prevalence rate, globalization, economic growth, and good governance on infant and child mortality rates in the selected African countries using sophisticated econometric methods. Our results reveal that an increase in public health expenditure, numbers of physicians, globalization, GDP growth and education would significantly reduce infant and child mortality rates. This study also reveals a significant positive relationship between the prevalence of HIV and infant and child mortality rates. These results can be considered as robust because (1) they pass all diagnostics tests, and (2) the good governance variable is considered as a controlled variable in the models, which is likely to reduce any bias in the coefficients that can be caused in the absence of a controlled variable. The findings of this study re-emphasise the importance of increased public health expenditure, numbers of physicians/1000 population, GDP growth rate, literacy rate, globalization, and the maintenance of good governance in reducing infant and child mortality rates in African countries. The effectiveness of some of these factors such as GDP growth and public health expenditure depends heavily on good decision making and implementation of decisions; therefore, policy makers should pay attention to improve good governance in the country. A striking result reveal from this study is that prevalence of HIV significantly increases infant and child mortality rates. We know that the prevalence of HIV is very high in African countries; therefore, governments and policy makers have no other alternative but to control the prevalence of HIV to reduce infant and child mortality rates from the African region. This is a big task but is of vital importance. Globalization is also found as another novel impetus to facilitate more global amenities and medical facilities for ensuring better infant and child health. Therefore, African countries should embrace globalization as an opportunity, and the necessary policy formulations and their execution to exploit more benefits from globalization are a time demanded issue.

However, globalisation has different aspects; hence these countries should be careful too. Economic globalization improves the efficiency of enterprises and plays an important role in increasing the size of the economy. It involves long-distance flows of goods, services and capital and the information and perceptions that accompany market exchange. It is generally welfare improving as increased income can be spent in health sector as well; however, since all the countries under study are developing/poor countries, open acceptance of rapid globalisation may adversely impact the infant industries of these countries, as they are not globally competitive.

Globalization also impacts the life and work of people, their families, and societies through employment, working conditions, income and social protection. Globalisation also affects the culture of a country too. Countries under study must be careful to grab the positive aspects of globalisation to improve the living condition of their people.

Globalisation also intensifies and expands political interrelations across the globe. There are growing influence of international organizations such as the United Nations, World Bank and World Health Organisation on the actions of countries. These organisations work as a development partner of a country to enhance the living standard and life expectancy of general population.

There are some limitations in this study. We cannot include many African countries in our study due to unavailability of the data on selected variables. Thus, our study is limited to a panel of 14 countries only. Future research is recommended to consider more countries/all countries in the region along with other relevant variables such as disease type, corruption, gender roles, and inequality with complete and updated data periods.

## Data Availability

Data used in this study are collected from the World Development Indicator [87] and World Governance Indicator [88] of the World Bank, and Human Development Reports [32] of the United Nation Development Program (UNDP). The complete structured data set used for the study may be supplied by the corresponding author on reasonable request.

## Footnotes

1 Nurses/midwives (or both) may also be used instead of physicians; however data on these variables are not available for all selected years for all sample countries.

2 Women’s education or investments and incentives for girls’ education are not used because of lack of data.

## References

[1] Akinci F, Hamidi S, Suvankulov F, Akhmedjonov A. Examining the impact of health care expenditures on health outcomes in the middle east and N. Africa. J Health Care Finance. 2014;41(1):1–23.

[2] Akinkugbe O, Mohanoe M (2009). Public health expenditure as a determinant of health status in Lesotho. Soc Work Public Health.

[3] Akinlo AE, Sulola AO (2019). Health care expenditure and infant mortality in sub-Saharan Africa. J Policy Model.

[4] Alonso JM, Clifton J, Díaz-Fuentes D (2017). The impact of government outsourcing on public spending: evidence from European Union countries. J Policy Model.

[5] Anwar A, Ayub M, Khan N, Flahault A (2019). Nexus between air pollution and neonatal deaths: a case of Asian countries. Int J Environ Res Public Health.

[6] Araujo JA, Silva AF, Wichmann R (2015). Impact of poverty on multidimensional infant mortality rate in Brazil. Value Health.

[7] Arunda MO, Choudhry V, Ekman B, Asamoah BO (2016). Under-five mortality and maternal HIV status in Tanzania: analysis of trends between 2003 and 2012 using AIDS Indicator survey data. Glob Health Action.

[8] Asumadu-Sarkodie S, Owusu PA (2016). The casual nexus between child mortality rate, fertility rate, GDP, household final consumption expenditure, and food production index. Cogent Econ Finance.

[9] Alemu AM. To what extent does access to improved sanitation explain the observed differences in infant mortality in Africa? Afr J Prim Health Care Fam Med. 2017;9(1). 10.4102/phcfm.v9i1.1370. 10.4102/phcfm.v9i1.1370 PMC5458576 28582990

[10] Anyanwu JC, Erhijakpor AE (2009). Health expenditures and health outcomes in Africa. Afr Dev Rev.

[11] Bailey D, Katz JN (2011). Implementing panel corrected standard errors in R: the pcse package. J Stat Softw.

[12] Azarnert LV (2006). Child mortality, Fertility, and human capital accumulation. J Popul Econ.

[13] Baum CF (2001). Residual diagnostics for cross-section time series regression models. Stata J.

[14] Beck N, Katz JN (1995). What to do (and not to do) with time-series cross-section data. Am Polit Sci Rev.

[15] Becker GS (1964). Human capital: a theoretical and empirical analysis, with special reference to education.

[16] Boachie MK, Ramu K (2016). Effect of public health expenditure on health status in Ghana. Int J Health.

[17] Cameron AC, Trivedi PK (2009). Microeconometrics: methods and applications.

[18] CIA (2020). World Factbook.

[19] Chireshe J, Ocran MK (2020). Health care expenditure and health outcomes in sub-Saharan African countries. Afr Dev Rev.

[20] Dutta UP, Gupta H, Sarkar AK, Sengupta PP (2020). Some determinants of infant mortality rate in SAARC countries: an empirical assessment through panel data analysis. Child Indic Res.

[21] Edeme KR (2017). Public health expenditure and health outcomes in Nigeria. Am J Biomed Life Sci.

[22] Emamgholipour S, Asemane Z (2016). Effect of governance indicators on under-five mortality in OECD nations: generalized method of moments. Electron Physician.

[23] Ensor T, Cooper S, Davidson L, Fitzmaurice A, Graham WJ (2010). The impact of economic recession on maternal and infant mortality: lessons from history. BMC Public Health.

[24] Farag M, Nandakumar AK, Wallack S, Hodgkin D, Gaumer G, Erbil C (2012). Health expenditures, health outcomes and the role of good governance. Int J Health Care Finance Econ.

[25] Farahani, M., Subramanian, S.V., & Canning, D.. Short and long-term relationship between physician density on infant mortality: a longitudinal econometric analysis. PGDA working paper no. 49. National Institute on Aging. 2009.

[26] Furnee CA, Groot W, van den Brink HM (2008). The health effects of education: a meta-analysis. Eur J Pub Health.

[27] Gakidou E, Cowling K, Lozano R, Murray CJ (2010). Increased educational attainment and its effect on child mortality in 175 countries between 1970 and 2009: a systematic analysis. Lancet.

[28] Garrib A, Jaffar S, Knight S, Bradshaw D, Bennish ML (2006). Rates and causes of child mortality in an area of high HIV prevalence in rural South Africa. Tropical Med Int Health.

[29] Golinelli D, Toscano F, Bucci A, Lenzi J, Fantini MP, Nante N, Messina G (2017). Health expenditure and all-cause mortality in the ‘galaxy’ of Italian regional healthcare systems: a 15-year panel data analysis. Appl Health Econ Health Policy.

[30] Granger CWJ (1969). Investigating causal relations by econometric models and cross-spectral methods. Econometrica.

[31] Grossman M (1972). On the concept of health capital and the demand for health. J Polit Econ.

[32] HDR (2020). Human development reports.

[33] Hlafa B, Sibanda K, Hompashe DM (2019). The impact of public health expenditure on health outcomes in South Africa. Int J Environ Res Public Health.

[34] Houweling TAJ, Kunst AE, Looman CWN, Mackenbach JP (2005). Determinants of under-5 mortality among the poor and the rich: a cross-national analysis of 43 developing countries. Int J Epidemiol.

[35] Ikpesu F, Vincent O, Dakare O (2019). Growth effect of trade and investment in sub-Saharan Africa countries: empirical insight from panel corrected standard error (PCSE) technique. Cog Econ Finance.

[36] Jani VJ, Joshi NA, Mehta DJ (2019). Globalization and health: an empirical investigation. Global Soc Policy.

[37] Jebeli SSH, Hadian M, Souresrafil A (2019). Study of health resource and health outcomes: organization of economic corporation and development panel data analysis. J Educ Health Promot.

[38] Jönsson K (2005). Cross-sectional dependency and size distortion in a small-sample homogeneous panel data unit root test. Oxf Bull Econ Stat.

[39] Kanmiki EW, Bawah AA, Agorinya I, Achana FS, Awoonor-williams JK, Oduro AR, Phillips JF, Akazili J (2014). Socio-economic and demographic determinants of under-five mortality in rural northern Ghana. BMC Int Health Hum Rights.

[40] Karyani AK, Kazemi Z, Shaahmadi F, Arefi Z, Meshkani Z (2015). The Main determinants of under 5 mortality rate (U5MR) in OECD countries: a cross-sectional study. Int J Paediatr.

[41] Keats, A. Women’s schooling, fertility, and child health outcomes: Evidence from Uganda’s free primary education program. J Develop Econ. 2018;135:142–59. 10.1016/j.jdeveco.2018.07.0020.

[42] Kiros GE, Hogan DP (2001). War, famine and excess child mortality in Africa: the role of parental education. Int J Epidemiol.

[43] Kiross GT, Chojenta C, Barker D, Loxton D (2020). The effects of health expenditure on infant mortality in sub-Saharan Africa: evidence from panel data analysis. Heal Econ Rev.

[44] KOF Globalisation Index (2020). KOF globalisation index – KOF Swiss economic institute | ETH Zurich.

[45] Kousar S, Shabbir A, Shafqat R. Investigation of socioeconomic determinants on child death in south Asian countries: a panel Cointegration analysis. Omega (Westport). 2020. 10.1177/0030222820915023. 10.1177/0030222820915023 32276562

[46] Lallemant C, Halembokaka G, Baty G, Ngo-Giang-Huong N, Barin F, Le Coeur S (2010). Impact of HIV/Aids on child mortality before the highly active antiretroviral therapy era: a study in Pointe-Noire, republic of Congo. J Trop Med.

[47] Le T-H, Nguyen CP (2019). Is energy security a driver for economic growth? Evidence from a global sample. Energy Policy.

[48] Le T-H, Nguyen CP, Su TD, Tran-Nam B. The Kuznets curve for export diversification and income inequality: Evidence from a global sample. Economic Analysis and Policy. 2020;65:21–39.

[49] Liang S, Macinko J, Yue D, Meng Q (2019). The impact of the health care workforce on under-five mortality in rural China. Hum Resour Health.

[50] Liebert, H. & Mäder, B.. Physician density and infant mortality: a Semiparametric analysis of the returns to health care provision. CESifo working paper no. 7209. 2018, Available at SSRN: https://ssrn.com/abstract=3275382

[51] Magalhães, M. & Africano, A. P. A panel analysis of the FDI impact on international trade. FEP working papers 235, Universidade do Porto, Faculdade de Economia do Porto: NIPE - Universidade do Minho. 2007.

[52] Makochekanwa A, Madziwa C (2016). Impact of public health expenditure on health outcomes in Zimbabwe (1980-2014). Univ Zimbabwe Bus Rev.

[53] Manda DK, Mugo MG, Murunga J (2020). Health expenditures and health outcomes in Kenya. Eur Sci J.

[54] Marinda E, Humphrey JH, Iliff PJ, Mutasa K, Nathoo KJ, Piwoz EG, Moulton LH, Salama P, Ward BJ (2007). Child mortality according to maternal and infant HIV status in Zimbabwe. Pediatr Infect Dis J.

[55] Martens P, Akin SM, Maud H, Mohsin R (2010). Is globalization healthy: a statistical indicator analysis of the impacts of globalization on health. Glob Health.

[56] McAlister C, Baskett TF (2006). Female education and maternal mortality: a worldwide survey. J Obstet Gynaecol Can.

[57] Muldoon KA, Galway LP, Nakajima M, Kanters S, Hogg RS, Bendavid E, Mills EJ (2011). Health system determinants of infant, child and maternal mortality: a cross-sectional study of UN member countries. Glob Health.

[58] Nakiyingi JS, Bracher M, Whitworth JAG, Ruberantwari A, Busingye J, Mbulaiteye SM, Zaba B. Child survival in relation to mother's HIV infection and survival: Evidence from a Ugandan cohort study. AIDS (London, England). 2003;17:1827-34. 10.1097/01.aids.0000076274.54156.41. 10.1097/00002030-200308150-00012 12891069

[59] Newell M-L, Brahmbhatt H, Ghys PD (2004). Child mortality and HIV infection in Africa. AIDS.

[60] Nguea SM, Noumba I, Noula AG (2020). Does globalization improve health in SubSaharan African countries?. Econ Bull.

[61] Nicholas A, Edward NA, Bernardin S. The effect of health expenditure on selected maternal and child health outcomes in Sub-Saharan Africa. Int J Soc Econ. 2016;43(12):1386–99. 10.1108/ijse-08-2015-0199.

[62] Novignon J, Olakojo SA, Nonvignon J (2012). The effects of public and private health care expenditure on health status in sub-Saharan Africa: new evidence from panel data analysis. Heal Econ Rev.

[63] Novignon J, Lawanson AO (2017). Health expenditure and child health outcomes in Sub-Saharan Africa. Afr Rev Econ Finance.

[64] Nyamuranga C, Shin J (2019). Public health expenditure and child mortality in southern Africa. Int J Soc Econ.

[65] Olagunju KO, Ogunniyi AI, Oguntegbe KF, Raji IO, Ogundari K (2019). Welfare impact of globalization in developing countries: examining the mediating role of human capital. Economies.

[66] Olatunde OS, Adebayo AA, Fagbemi F. Health Expenditure and Child Health Outcome in West Africa. Int J Soc Sci Perspect. 2019;5(2):72–83. 10.33094/7.2017.2019.52.72.83.

[67] Oloo JA (2005). Child mortality in developing countries: challenges and policy options. East Afr Soc Sci Res Rev.

[68] Pérez-Moreno S, Blanco-Arana MC, Bárcena-Martín E (2016). Economic cycles and child mortality: a cross-national study of the least developed countries. Econ Hum Biol.

[69] Pesaran MH (2004). General diagnostic tests for cross section dependence in panels.

[70] Preble EA (1990). Impact of HIV/AIDS on African children. Soc Sci Med.

[71] Qiu J, Ma Q, Wu L (2019). A moving blocks empirical likelihood method for panel linear fixed effects models with serial correlations and cross-sectional dependences. Econ Model.

[72] Rahman MM, Khanam R, Rahman M (2018). Health care expenditure and health outcome nexus: new evidence from the SAARC-ASEAN region. Glob Health.

[73] Rahman MM, Alam K, Velayutham E. Is industrial pollution detrimental to public health? Evidence from the world’s most industrialised countries. BMC Public Health. 2021;21(1). 10.1186/s12889-021-11217-6. 10.1186/s12889-021-11217-6 PMC8213381 34144705

[74] Rahman MM, Alam K. The role of socio-economic and female indicators on child mortality rate in Bangladesh: a time series analysis. Omega (Westport). 2021;003022282199361. 10.1177/0030222821993616. 10.1177/0030222821993616 33567983

[75] Rahman MM, Alam K. The nexus between health status and health expenditure, energy consumption and environmental pollution: empirical evidence from SAARC-BIMSTEC regions. BMC Public Health. 2021a;21(1). 10.1186/s12889-021-11534-w. 10.1186/s12889-021-11534-w PMC8445613 34530797

[76] Revathy A, Paramasivam P (2018). Study on panel Cointegration, regression and causality analysis in papaya Markets of India. Int J Curr Microbiol App Sci.

[77] Russo LX, Scott A, Sivey P, Dias J (2019). Primary care physicians and infant mortality: evidence from Brazil. PLoS One.

[78] Salahuddin M, Vink N, Ralph N, Gow J (2020). Effects of economic growth, foreign direct investment and internet use on child health outcomes: empirical evidence from South Africa. Dev Stud Res.

[79] Seitaridis M, Koulakiotis A (2013). Unemployment and government expenditure in the Eurozone: a panel data analysis. Public Municipal Finance.

[80] Shahbaz M, Shafiullah M, Mahalik MK (2019). The dynamics of financial development, globalisation, economic growth and life expectancy in sub-Saharan Africa. Aust Econ Pap.

[81] Shetty A, Shetty S (2014). The impact of doctors per capita on the mortality rate in Asia. Int J Med Pharm Sci.

[82] Tlou B, Sartorius B, Tanser F (2018). Investigating risk factors for under-five mortality in an HIV hyper-endemic area of rural South Africa, from 2000–2014. PLoS One.

[83] Urquieta-Salomón J, Lamadrid-Figueroa H, Angeles G, Montoya A, Rojas-Martínez R, Martínez-Nolasco A, Torres-Pereda P, O’Shea G, Villagrán VM, Halley E, Delgado-Sánchez V, Lazcano-Ponce E (2020). Impact of the ‘Seguro Médico Siglo XXI’ medical insurance programme on neonatal and infant mortality in Mexico, 2006–14: an ecological approach to estimation. Health Policy Plan.

[84] UNDP (2015). Sustainable development goals (goal 3: good health and well-being).

[85] WHO (2015). Sustainable development goal 3: health.

[86] Wang L (2003). Determinants of child mortality in LDCs. Health Policy.

[87] WDI (2020). World development indicators.

[88] WGI (2020). World governance indicator.

[89] WHO (2020). Factors associated with trends ininfant and child mortality in developing countries during the 1990s. Bull World Health Organ.

[90] WHO (2020). Reducing child mortality in India in the new millennium. Bull World Health Organ.

[91] Welander A, Lyttkens C, Nilsson T. Globalization and child health in developing countries: the role of democracy. SSRN Electron J. 2014. 10.2139/ssrn.2469041.

[92] Wooldridge JM (2002). Econometric analysis of cross section and panel data.

[93] Yaya S, Ekholuenetale M, Tudeme G, Vaibhab S, Bishwajit G, Kadio B (2017). Prevalence and determinants of childhood mortality in Nigeria. BMC Public Health.

[94] Young F, Critchley JA, Johnstone LK, Unwin NC (2009). A review of co-morbidity between infectious and chronic disease in sub Saharan Africa: TB and diabetes mellitus, HIV and metabolic syndrome, and the impact of globalization. Glob Health.

